# Recurrent and Progressive Abdominal Pain and Enteritis in a Japanese Patient with Paroxysmal Nocturnal Hemoglobinuria

**DOI:** 10.1155/2014/310750

**Published:** 2014-01-27

**Authors:** Akihisa Hino, Yukiko Yamashita, Mitsuhiro Yamaguchi, Yasuhiko Azenishi

**Affiliations:** ^1^Department of Internal Medicine, Minoh City Hospital, 7-1 Kayano 5-chome, Minoh City, Osaka 562-0014, Japan; ^2^Department of Hematology and Chemotherapy, Osaka Medical Center for Cancer and Cardiovascular Diseases, 8-13F, 1-3-2 Nakamichi, Higashinari-ku, Osaka 537-8511, Japan

## Abstract

This case report describes a young male patient with recurrent abdominal pain persisting for more than 16 months. Clinical investigations showed signs of inflammation and pancytopenia. A diagnosis of paroxysmal nocturnal hemoglobinuria (PNH) was made 9 months after the onset of the abdominal pain, following endoscopic examinations that revealed evidence of a previously unknown hemorrhage. Regular monitoring indicated that the abdominal pain was associated with elevations in lactate dehydrogenase, C-reactive proteins, and D-dimer levels. The patient started treatment with the complement inhibitor eculizumab shortly after it was approved for use in Japanese PNH patients with hemolysis. Resolution of the abdominal pain and normalization of clinical parameters were noted within 3 weeks from treatment initiation.

## 1. Introduction

Paroxysmal nocturnal hemoglobinuria (PNH) is a rare and progressive hematopoietic stem cell disorder in which hematopoietic cells are deficient in glycosylphosphatidylinositol (GPI), which leads to a lack of the complement inhibitor proteins CD55 and CD59 on the surface of blood cells. This results in the characteristic intravascular hemolysis of red blood cells, along with platelet activation and aggregation [1]. PNH is fatal in up to 35% of patients within 5 years of diagnosis and in up to 50% of patients within 10 to 15 years of diagnosis [2, 3], largely as the result of thrombosis. Patients with PNH have an approximately 62-fold greater risk of thrombosis compared with the general population [4] and an estimated 10.2-fold increase in mortality risk [3]. The liver, kidney, brain, and gut are common sites of thromboembolism, and up to 44% of patients with PNH will experience a clinically evident thrombosis [3, 5]. Although the etiology and symptoms of PNH are similar in Japanese/Asian and Western patients, it has been suggested that the clinical course of the disease may differ between the ethnic groups [6]. Here, we describe a Japanese patient who experienced extensive abdominal pain over a period of 16 months accompanied by pancytopenia, signs of inflammation, and thickening of the intestinal wall.

## 2. Case Study

In May 2009, a 22-year-old male with no relevant personal or family medical history started experiencing lower abdominal pain lasting for several days with repeated periods of exacerbation and remission.

In December 2009, the patient attended an outpatient department for detailed examination, as the abdominal pain had not resolved. Routine tests revealed pancytopenia, with a white blood cell (WBC) count of 2,400/*μ*L (lower limit of normal (LLN) 3,800/*μ*L), a platelet count of 7.3 × 10^4^/*μ*L (LLN 15.0 × 10^4^/*μ*L), and a hemoglobin level of 10.8 g/dL (LLN 12.1 g/dL). The patient went to the emergency department 5 days later with abdominal pain that spread across the entire abdomen. Blood tests gave indications of inflammation with elevated C-reactive protein (CRP) (99.6 mg/L; upper limit of normal (ULN) 6 mg/L) and suspicions of enteritis. As a consequence, the patient was hospitalized. Further blood tests on admission showed that the platelet count was still low (7.5 × 10^4^/*μ*L) but the WBC count (8,200/*μ*L) had increased to within the normal range (3,800–9,800/*μ*L). However, fibrinogen degradation products (FDP), indicative of a thrombotic event, were significantly elevated (122 × 10^3^ mg/dL; ULN 0.4 × 10^3^ mg/dL).

Endoscopic analyses of the gastrointestinal (GI) tract showed no abnormalities in the esophagus, stomach, or lower GI tract. The duodenum, particularly at the proximal end, showed edema, inflammation, and reddening, with adhesion of necrotic matter. This was confirmed by pathological investigations, and a diagnosis of nonspecific mucosal inflammation was made. Further investigations of the small intestine showed segmental edematous mucosa and sporadic erosions that were several centimeters in size, although there was no evidence of ulceration or tumor development. In addition, evidence of an unidentified hemorrhage was seen.

**Figure 1:**
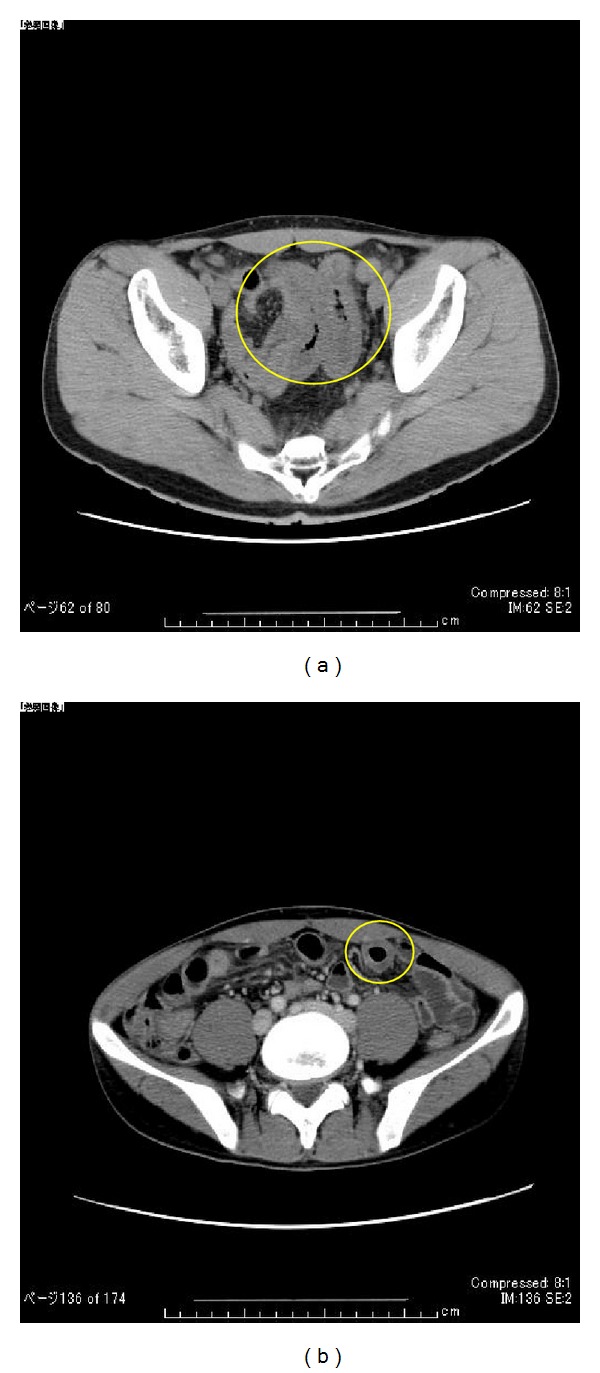
(a) CT scan in December 2009; thickening of small intestine wall is circled. (b) Contrast-enhanced CT scan in February 2010; edema-like contrast effect of intestinal wall thickening is circled.

A computerized tomography (CT) abdominal scan revealed intermittent (full thickness) thickening of the small intestinal wall (Figure 1(a)). Two months later, in February 2010, a follow-up, contrast-enhanced CT scan showed a different distribution of intestinal thickening, with an edema-like contrast effect seen in the thickened intestinal tract (Figure 1(b)).

Bone marrow investigations, also performed in December 2009, proved to be normal, although the patient did have an elevated serum lactate hydrogenase (LDH) level of 702 U/L (ULN 200 U/L) and a haptoglobin concentration of <10 mg/dL, suggestive of hemolytic anemia. In light of these findings, tests for PNH were carried out. Both a direct and indirect Coombs test proved negative, although a sugar water test was positive. A diagnosis of PNH was confirmed using flow cytometry, which showed a neutrophil clone size of 57% and a red blood cell clone size of 9%. An assessment of quality of life was performed using the FACIT-Fatigue instrument [7]. The patient had a score of 20/52 at diagnosis of PNH, which is indicative of severe fatigue.

**Figure 2:**
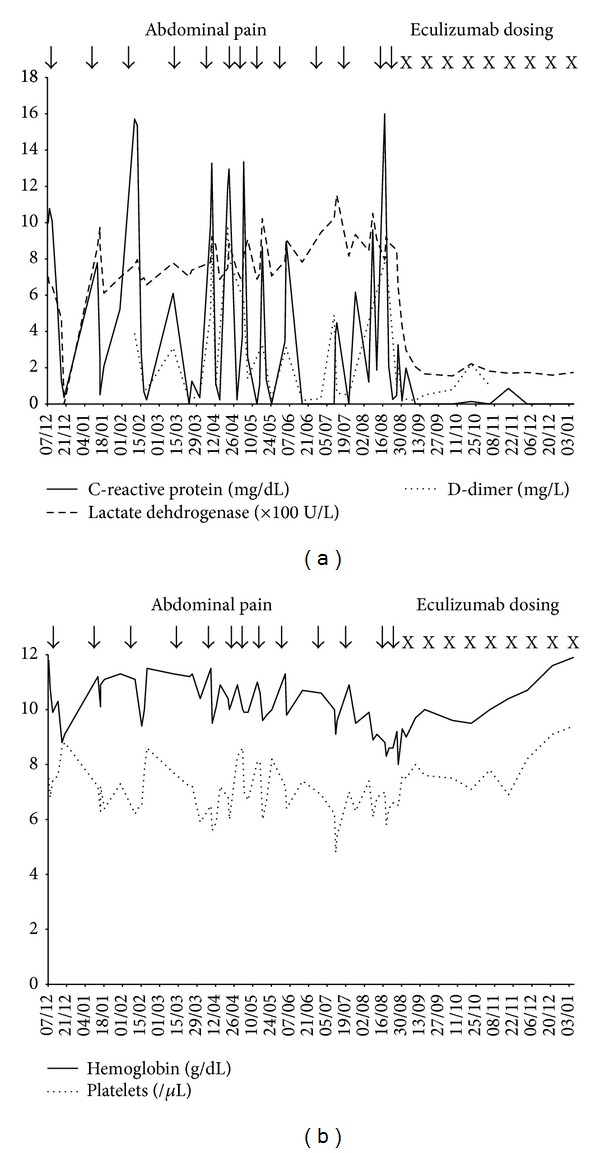
Clinical course from December 2009 to January 2011. (a) C-reactive protein, lactate dehydrogenase, and D-dimer levels. (b) Hemoglobin and platelet levels.

Gastrointestinal examinations to determine the cause of the abdominal pain were inconclusive, although it was suspected that the enteritis may have been caused by thrombosis. Accordingly, prophylaxis with oral administration of warfarin (1–3 mg/day) was initiated. Two weeks after hospital admission, the patient was discharged with the abdominal pain and inflammatory reactions in remission after further treatment with metronidazole, fasting, and fluid replacement. Between the end of April and the end of August 2010, despite continued warfarin administration, the patient experienced recurrent abdominal pain that was occasionally of sufficient severity to require hospitalization. Routine assessments of hemoglobin, CRP, platelets, D-dimer, and LDH were collected over this period and are plotted along with the occurrence of abdominal pain in Figure 2(a). However, serum creatinine level was within normal range.

From Figure 2(a), it can be seen that the periods of abdominal pain were clearly associated with increases in CRP and D-dimer, indicators of inflammation and thrombosis, respectively. The abdominal pain was also associated with peaks in LDH concentrations, which were generally 4-fold higher than the ULN, as well as decreases in levels of hemoglobin and platelets (Figure 2(b)). This clearly indicates that the enteritis experienced by the patient occurred at the same time as hemolytic episodes and that prophylactic administration of warfarin had no effect on the occurrence of enteritis, hemolysis, or thrombosis.

In June 2010, the humanized monoclonal antibody eculizumab was approved in Japan for the treatment of hemolysis in PNH patients. The patient was considered a suitable candidate for eculizumab therapy, and towards the end of August 2010, 2 weeks after vaccination with a meningococcal vaccine, administration of eculizumab was started. The initiation of treatment coincided with the beginning of a period of abdominal pain which, by the following day, had resolved (Figures 2(a) and 2(b)).

By the end of the first 3 weeks of eculizumab treatment, the abdominal pain and enteritis had completely resolved, levels of CRP had fallen to zero, LDH concentration and D-dimer were within the normal range (Figure 2(a)), and levels of platelets and hemoglobin were increasing (Figure 2(b)). These improvements were sustained with continued use of eculizumab out to the last assessments recorded in January 2011. Other assessments at this time showed no impairment in renal function. In December 2010, reassessment of the patient's quality of life showed a remarkable improvement, with FACIT-Fatigue scores increasing by 30 points (to 50/52), indicating that the patient had only minimal fatigue. The patient did not experience any significant adverse events during eculizumab treatment.

## 3. Conclusions

This case study details a patient with only mild anemia but repeated bouts of abdominal pain following periods of elevated hemolysis. The abdominal pain in this patient was assumed to be caused by the appearance of transient thrombi in intestinal microvessels, which induced intestinal edema. Administration of warfarin was insufficient to prevent the formation of these thrombi, as evidenced by the continued low platelet levels and increased FDP and D-dimer levels, as well as persistence of the abdominal pain. A previous case report demonstrated that recurrent ischemia of the small bowel can occur in PNH [8], a condition frequently associated with abdominal pain [9, 10]. In addition, it has been shown that those patients with PNH who experience abdominal pain have a 3.6-fold increased risk of thrombosis and 2.2-fold risk of premature mortality [11].

Hemolysis caused by PNH releases excess hemoglobin into the blood stream, leading to the consumption of endothelium-derived nitric oxide at a rate of 600-fold faster than normal [12, 13]. The depletion of nitric oxide causes the constriction of smooth muscle fibers, resulting in abdominal pain and vasoconstriction with decreased blood flow [9, 10], as well as increasing platelet activation and aggregation, endothelial swelling, and impaired fibrinolysis [14–16], all symptoms observed in this patient prior to the diagnosis of PNH. It has been reported that treatment with eculizumab reduces endothelial activation and markers of thrombin generation, further demonstrating the association between an inflammatory, prothrombotic state and chronic complement activation in PNH patients [17]. Interestingly, the same study reported CRP levels within the normal range in 83% of the patients. We demonstrated that CRP levels increased above normal during episodes of abdominal pain and returned to normal when the abdominal pain resolved. These data suggest that markers such as CRP may be effective short-term measures of thrombosis risk despite the absence of obvious clinical evidence of a thrombosis. Future studies measuring CRP levels during presentation of hemolytic symptoms such as abdominal pain may provide further evidence of PNH patients having underlying thrombogenesis.

This diagnosis of PNH was not confirmed until approximately 9 months after the patient's first clinical investigations, which were associated with complement-mediated inflammation. Administration of warfarin failed to prevent recurrence of the abdominal pain and underlying enteritis while serum LDH and haptoglobin profiles indicated hemolytic anemia. These clinical observations prompted the flow cytometric testing for PNH despite a normal bone marrow profile [18]. Following confirmation of PNH and the initiation of therapy with eculizumab, hemolysis was significantly reduced, the hemolysis-mediated abdominal pain resolved completely, and the patient no longer suffered from the severe fatigue that had previously restricted his activities. Given the serious and often fatal consequences of PNH, this case emphasizes the importance of conducting a full clinical assessment and recognizing abdominal pain as a risk factor for thrombotic events in patients with PNH.
